# Optimization of stimulation parameters for epi-retinal implant based on biosafety consideration

**DOI:** 10.1371/journal.pone.0236176

**Published:** 2020-07-22

**Authors:** Yijie Lu, Shan Qin, Lei Zhao, Lan Yue, Tianzhun Wu, Bo Qin, Zhen Xu

**Affiliations:** 1 Shenzhen Institutes of Advanced Technology, Chinese Academy of Sciences, Shenzhen, China; 2 Shenzhen Aier Eye Hospital, Shenzhen, China; 3 Shenzhen Shekou People’s Hospital, The Second Xiangya Hospital of Central South University Shenzhen Hospital, Shenzhen, China; 4 Roski Eye Institute, University of Southern California, Los Angeles, California, United States of America; Shanghai Mental Health Center, CHINA

## Abstract

**Background:**

Optimizing stimulation protocol is essential for clinical application of retinal prosthesis. Elongating stimulation pulse width (~25ms /phase) has been proposed as an effective method to improve spatial resolution of epi-retinal implants. However, it is unknown whether longer stimulus pulse width will increase the risk of damaging the retina. In addition, with the advent of next generation retinal prosthesis featuring high-density microelectrode array, it is tempting to optimizing a single set of parameters for all electrodes instead of optimizing parameters of each electrode, but this approach raised biosafety concern. We sought to study the effect of stimulus pulse width on the response of retinal ganglion cells to electrical stimulation, and evaluate if the single parameter set approach was valid based on biosafety measures.

**Methods:**

We stimulated mouse retina using biphasic pulse waveform generated by chosen electrodes (single or a 3x3 assembly) from multiple microelectrode arrays, recorded their action potentials and performed spike sorting. We tested various stimulus intensity with two fixed pulse width: a short one for 1 millisecond per phase, and a long one for 25 milliseconds per phase. All these assays were performed on two mouse models: the wildtype C57BL/6J mice and the photoreceptor degenerated rd10 mice. The action-potential-frequency vs stimulus amplitude profiles were plotted, and three parameters were extracted: the threshold (the lowest stimulus amplitude activating RGC units), safety-limit (stimulus amplitude that attenuated the firing rate to half of the maximum response), and the stimulation amplitude range (the difference between threshold and safety limit parameters).

**Results:**

In single-electrode stimulation experiment, we found that on average 85% of the recorded units showed attenuated response to extreme stimulation; among those units, an average of 51% stopped responding during stimulation ramping and failed to recover after one-hour post-stimulation, indicating extreme stimulation can damage RGC units. Twenty-five-millisecond pulse stimulation significantly reduced safety-limit and stimulation-amplitude-range parameters of recorded RGC units compared to 1ms pulse stimulation. During stimulus amplitude ramping, the maximum proportion of responsive healthy RGC units was 51% on average in 25ms pulse condition, and 76% on average in 1ms pulse condition, indicating long pulse may inflict more strain on RGCs, and a significant amount of inappropriately stimulated RGCs always exist. The contrast of these proportions could be explained by the tight correlation between the threshold and safety-limit parameter in 25ms pulse condition. These results were corroborated by those from 3x3 array stimulation experiments.

**Conclusion:**

Base on a biosafety measure (RGCs’ evoked firing rate in response to electrical stimulation), we proposed that longer stimulation pulse width could lead to reduced retinal response and thus highlighted the importance of carefully setting the stimulation amplitude in this case. Our results also suggested that optimizing a single set of parameters for all electrodes without individual tweaking always generated a significant amount of inappropriately stimulated RGCs, especially in the long pulse stimulation condition.

## Introduction

The retinal photoreceptors are the primary neural cells that transform light into graded electrical signals. With accelerating aging population, blindness diseases with symptoms of photoreceptor degeneration, such as age-related macular degeneration, are showing higher incidence, and pose severe challenges to public health management. In lots of cases, the inner retina, especially the relay to the brain—the retinal ganglion cells (RGC), are largely spared in many such diseases [1, 2], and provide the possibility for retinal prosthesis to help patients with photoreceptor degeneration.

One core component of retinal prosthesis is the microelectrode array (MEA). The stimulation protocol largely determines the efficacy of implanted epi-retinal prosthesis. Long stimulation pulse width (25ms per phase) was considered vital for improving spatial resolution of epi-retinal prosthesis stimulation [3], but how elongation of stimulation pulse width will affect the risk of damaging retina is not known. According to strength-duration curve of typical neuron stimulation (e.g., [4]), for safety concern, elongation of pulse width needs compensation by reduction of the stimulus amplitude; therefore, the threshold amplitude of RGC activation by long pulse-width stimulation will also be reduced. However, the strength-pulse width curve of RGC stimulation shows that stimulus pulse width beyond 500μs hardly affected stimulus threshold amplitude [5]. In this case, it is possible that under long pulse condition, casually chosen stimulus amplitude well beyond threshold will easily damage retinas. Such risk has not been systematically studied. Also, photoreceptor degeneration was reported to significantly alter the threshold of RGC activation [6], and it is clinically relevant to investigate how RGCs respond to elongated stimulation pulse-width in photoreceptor degeneration condition.

Besides the above concern, with the development of retinal prosthesis featuring high-density microelectrode array, the electrode number reached about ten thousand, and the task of optimization will become extremely consuming. In this scenario, it is desirable to optimize only one set of unifying stimulation parameters for all electrode instead of tens of thousands of separate parameters for each electrode. However, this approach raises biosafety and stimulation efficiency concern, and needs validation.

In this study, we sought to evaluate the effect of stimulus pulse width on the response of RGCs to electrical stimulation, and investigate the risk of stimulus pulse-width elongation by monitoring the firing rate of stimulated RGCs. We also tried to verify the cons of optimizing one set of stimulus parameters for all electrodes in epi-retinal stimulation. To simulate the photoreceptor degeneration condition, the *Pde6b*^*rd10/rd10*^ (abbreviated rd10 hereafter) mice were also used in our experiments.

## Materials and methods

The experiment design was described in S1 Fig.

### Mice and retina isolation

Three-month old C57BL/6J wildtype mice and rd10 mice (Jax lab Stock No: 004297) reared with normal visual experience (12 h light/dark cycle) were used in the study. Formal approval to conduct animal experiments described in the article had been obtained from the Shenzhen Institute of Advanced Technology, Chinese Academy of Sciences. Use and handling of animals were strictly in accordance with the National Institute of Health Guide for the Care and Use of Laboratory Animals (NIH Publications No. 80–23) and approved by Institutional Animal Care and Use Committee of Shenzhen Institution of Advanced Technology, Chinese Academy of Sciences (approval number SIAT-IRB-180301-YGS-XUZHEN-A0244). Animals were dark adapted for at least 1 h, deeply anaesthetized with an i.p. injection of a mixture of ketamine (50 mg/kg) and xylazine (10 mg/kg), and decapitated. The eyes were immediately enucleated under very dim red light, and the retina was carefully dissected from the pigment epithelium in Ames’ medium equilibrated with 95% O2 and 5% CO2, cut into four pieces, and flat mounted, ganglion cell layer up, on a piece of membrane filter (AABP02500, Millipore, Billerica, MA). The membrane was then inverted onto a microelectrode array (120MEA100/30iR-ITO-gr, Multichannel System, Reutlingen, BW), with the ganglion cells from the mid-third area of retinas directly contacting the electrodes. We totally used 17 retinas from 17 mice: 4 retinas were from wildtype mice for 1ms pulse width stimulation, 4 retinas were from wildtype mice for 25ms pulse width stimulation, 4 retinas were from rd10 mice for 1ms pulse width stimulation, and 5 retinas were from 5 rd10 mice for 25ms pulse width stimulation.

### Microelectrode array stimulation and recording

The microelectrode array with retina attached was installed on the MEA2100 system (Multichannel System, Reutlingen, BW). The retina preparation was continuously perfused with oxygenated bicarbonate-buffered Ames’ medium at 35°C. Recording and stimulation were controlled by MC_Rack software (Multichannel System, Reutlingen, BW). Extracellular potential data were recorded from all 120 electrodes, with a sampling rate of 10 kHz, band-passed between 200~2000 Hz, and stored on a personal computer for offline analysis. A biphasic electrical pulse was delivered via one chosen electrode, or via a 3×3 electrode array chosen from the 120 electrodes. The discrete stimulation voltage amplitudes were from 0.15V to 0.27V with a 0.15V step. The pulse consisted of a cathodic (negative) voltage pulse of amplitude A and pulse width d, followed immediately by an anodic (positive) pulse of amplitude A/2 and pulse width 2d. Reported voltage values always referred to the negative phase amplitude A. Pulse width d was 1ms or 25ms, and always referred to the pulse width of the cathodic phase. The choice of pulse width was based on protocol from the previous study [3] (25ms) or our pilot study (1ms). A single pulse was used to stimulate RGCs and repeat for 20 times, and the interval between each pulse was 10s to allow full recovery of the stimulated RGCs. A 100ms pre-stimulus trace were recorded for each unit as a baseline of action potential firing rate, and a 1s post-stimulus trace were recorded for each unit as the response. The spiking activities of stimulated RGCs were checked one hour after the last round of stimulation, and the number of RGCs without any recovered activity were recorded.

### Offline-data processing

The action potentials recorded from the stimulating electrodes (single-electrode stimulation experiments) or center electrodes (3×3 electrode array stimulation experiments) were sorted into individual units by Offline Sorter (Plexon Inc) using principle component analysis algorithm. Offline data analysis (spike binning and aligning to stimulus) was done using Spike2 (Cambridge Electronic Design Limited), and plotted with Prism 7 (GraphPad Software, San Diego, CA). To screen for stable responsive unit activated by a given stimulation, we set the following criteria for RGC activation: 1) the action potential firing rate post-stimulation is at least 3 times greater than baseline; 2) for the 20-times repeated stimulations, the unit must meet criteria 1 for at least 10 times. We calculated the difference between the firing rate of the initial 300ms right after stimulation and that of the pre-stimulation baseline, and defined it as the evoked response. The action-potential-frequency vs stimulus-amplitude profiles were plotted, and three parameters were extracted: the threshold, safety limit and stimulation amplitude range. The definition of these key parameters can be seen in Fig 2A. The threshold was the lowest stimulus amplitude needed to activate the RGC, which is to elicit a firing rate three times larger than that of the baseline. The firing rate reached a maximum value as the stimulus amplitude increased. At certain stimulus amplitude, the firing rate decreased to less than half of the maximum response. By interpolation, we calculated the stimulus amplitude that attenuated the firing rate to half of the maximum response, and defined it as the safety limit. Stimulation amplitude range was defined as the difference between safety limit and threshold. We defined responsive healthy RGC units as those with threshold parameters lower than the stimulus amplitude being tested and safety limit parameters higher than the stimulus amplitude being test. Using scatter plots based on threshold and safety limit, we determined the number of responsive healthy RGC units. The proportion of responsive healthy RGC units in each experiment group were plotted against increasing stimulus amplitude, and its peak were determined.

### Statistical method

The distributions of threshold, safety limit and stimulation amplitude range from different groups of RGC units were compared via Kruskal-Wallis test and Dunn’s multiple comparison test (significance level was 0.05). To compare the scatter plot of threshold vs safety limit for RGC units under short pulse and long pulse condition, kernel-density based two sample comparison test were performed (significance level was 0.05). Furthermore, kernel-density based local two sample comparison test were performed to search for regions showing different scatter patterns that reached significance level (0,05), with a grid size of 0.15×0.15V. To Compare the proportion of healthy responsive units under different stimulation amplitude, multiple t-test were performed via false discovery rate approach, with the two-stage step-up method of Benjamini, Krieger and Yekutieli [7] (FDR = 1%). The correlation coefficients between threshold and safety limit for each group were determined by linear regression and Spearman ranking method. To detect differences between these correlation coefficient, Fisher transformation were performed on the coefficients, and the resulting Z-scores were compared under normal distribution (N(0,1)) assumption (significance level was 0.05)

## Results

**Fig 1:**
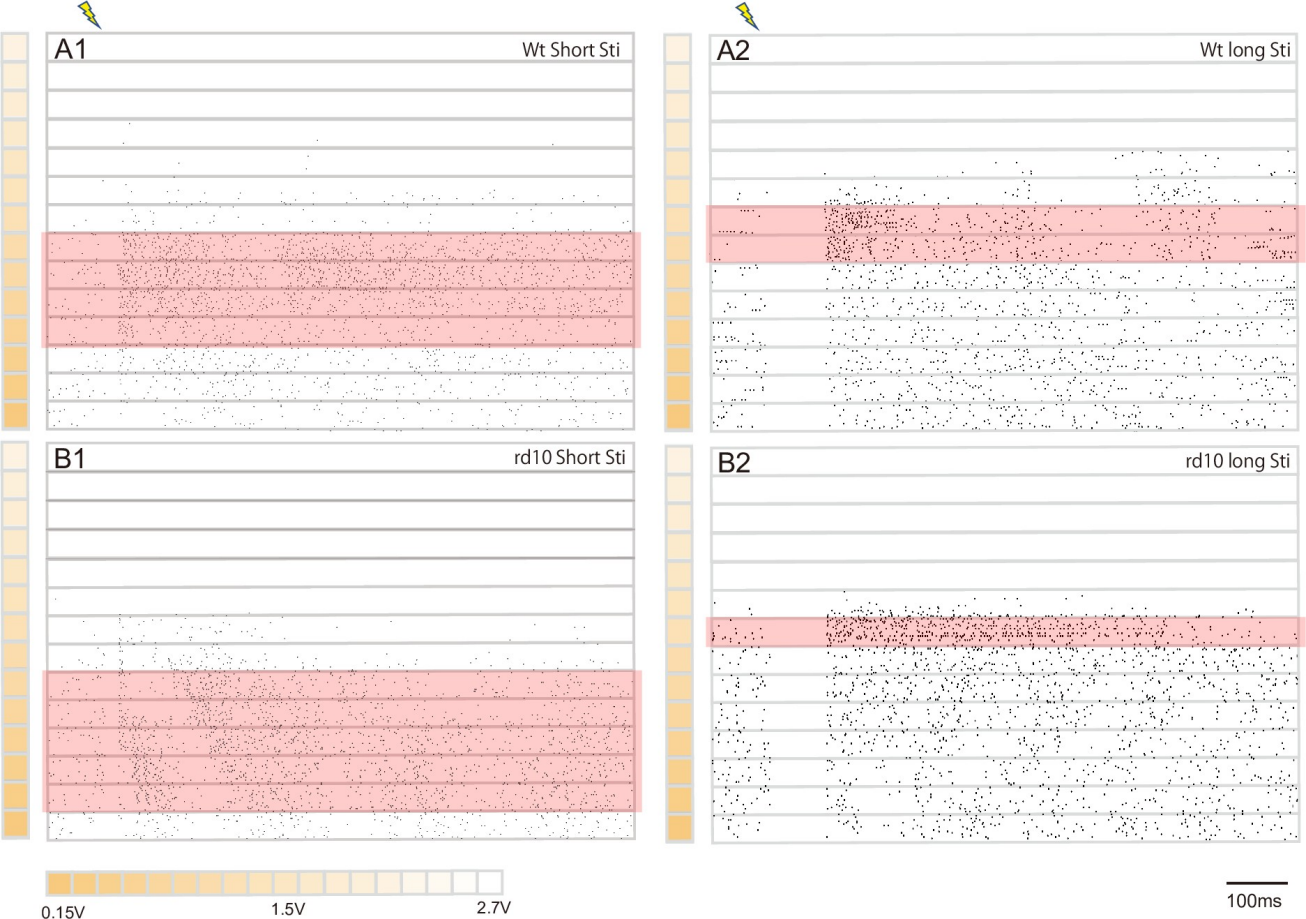
RGCs’ spiking response to electrical stimulation diminished after stimulus amplitude reached beyond certain limit. (A1) typical raster plot for wildtype RGCs evoked by uprising electrical stimulation with 1ms pulse-width. (A2) typical raster plot for wildtype RGCs evoked by uprising electrical stimulation with 25ms pulse-width. (B1) typical raster plot for rd10 RGCs evoked by uprising electrical stimulation with 1ms pulse-width. (B2) typical raster plot for rd10 RGCs evoked by uprising electrical stimulation with 25ms pulse-width. The lightening icon indicated the initiation of the stimulation, and the red rectangles indicated the stimulus amplitude range that effectively activated RGCs (see “Material and methods” for definition of RGC activation). The orange heat map indicated the stimulation amplitude.

Previously, long stimulus pulse width (~25 ms per phase) has been used to improve the spatial resolution of electrical stimulation of epi-retinal implants. To investigate whether and how long stimulus pulse width affected the health of RGCs—in either wildtype mice or rd10 mice—in comparison to short stimulus pulse width, we stimulated RGCs with biphasic electrical pulse delivered by a chosen electrode and analyzed signals recorded from the stimulating electrode. The stimulation pulse assumed a range of amplitude and two fixed stimulation pulse width: 1 ms/phase and 25 ms/phase. 133 units and 115 units was recorded for short stimulation and long stimulation in wild type mice retina, and 128 units and 153 units was recorded for short stimulation and long stimulation in rd10 mice retina. We found that the majority of recorded units first increased their firing rate in response to elevated stimulus amplitude, but then decreased their firing rate after the stimulus amplitude reached certain value (Fig 1A1, 1A2, 1B1 and 1B2). This was the case for both wildtype mouse retina and rd10 mouse retina, tested under both of the stimulation pulse width conditions: for short pulse, 84% (112/133) of the wildtype units and 81% (104/128) of the rd10 units showed such profile; for long pulse, 91% (105/115) of the wildtype units and 85% (130/153) of the rd10 units showed such profile. These attenuating units were used for later analysis. Moreover, high proportion (51%) of the above-mentioned units stopped responding during stimulation ramping and failed to recovery after one-hour post-stimulation. For short pulse, the proportion was 37% (42/112) in wild type retinas and 36% (38/104) in rd10 mouse retinas respectively, while for long pulse it was 68% (71/105) in wild type retinas and 62% (81/130) in rd10 mouse retina respectively (Table 1). This phenomenon indicated that extreme large stimulus intensity adversely affected the health of RGCs.

**Table 1:** Number of RGC units from different combination of mouse strain and stimulation pulse width.

		Stimulus pulse width	
		*1 ms*	*25 ms*
**Mouse strain**	*wildtype*	total unit number: **133**	total unit number: **115**
		units with attenuated response: **112**	units with attenuated response: **105**
		units failing to recover: **42**	units failing to recover: **71**
	*rd10*	total unit number: **128**	total unit number: 153
		units with attenuated response: **104**	units with attenuated response: **130**
		units failing to recover: **38**	units failing to recover: **81**

A large proportion of the units failed to recover after being over-stimulated.

**Fig 2:**
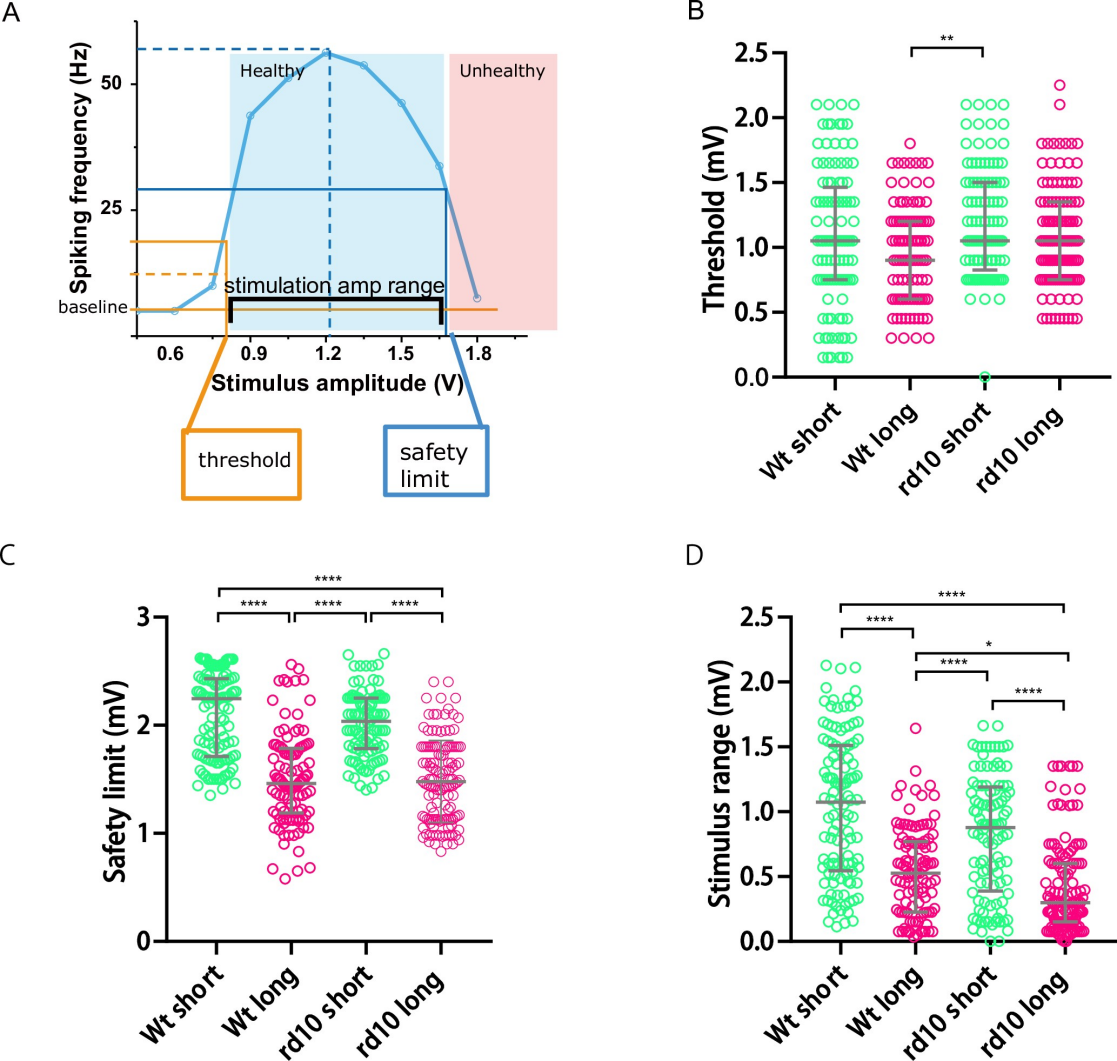
Significant differences existed in the distributions of threshold, safety limits and stimulation amplitude range of the recorded RGC units. (A) definition of RGC threshold, safety limit and stimulation amplitude range of the recorded RGC unit. (B) distribution of threshold for recorded RGC units. (C) distribution of safety limit for recorded RGC units. (D) distribution of stimulation amplitude range for recorded RGC units. The grey lines was at median with interquartile range. *, p<0.05; **, p<0.0021; ***, p<0.0002; ****, p<0.0001; statistics were performed via Kruskal-Wallis test and Dunn’s multiple comparison test (see statistic result in S1–S3 Tables).

We plotted RGCs’ action potential firing rate against the corresponding stimulus amplitude. From the profiles, we extracted several feature parameters for each RGC (Fig 2A). We defined the lowest stimulus amplitude that can activate an RGC to be its threshold. Since most RGCs attenuated their response to extreme large stimulation intensity, to find an indicator of the maximum stimulus intensity an RGC can withstand, we determined the stimulus amplitude that attenuated the recorded units’ firing rate to half of their maximum response by interpolation, and defined such amplitudes as the units’ safety limit. The safety limit parameter was used as the boundary that stimulus amplitude could not surpass. To reveal the relationship between threshold and safety limit parameter, we calculated their difference and defined it as the stimulation amplitude range. We calculate the above-mentioned parameters for each recorded RGC unit (Fig 2B, 2C and 2D). Although we did detect significant differences among thresholds of different groups, due to the large variations, it was difficult to determine the optimal stimulus amplitude (Fig 2B). However, longer stimulus pulse width did significantly and consistently lower the safety limit parameters (Fig 2C), which indicated that long pulse stimulation made the activated RGCs more vulnerable to high stimulus amplitude. In addition, when we pooled the stimulation-amplitude-range parameters for all of the recorded units, it was obvious that long pulse stimulation greatly compressed the safe stimulation-amplitude-range of RGC units (Fig 2D). This phenomenon was especially conspicuous for the rd10 retina: the median of the safe stimulation range under long pulse condition was only 0.3V, while the median of the safe stimulation range under short pulse condition was 0.88V. The compression of safe stimulation-amplitude-range implicated that the operation window for parameter adjustment under long pulse condition was severely limited.

**Fig 3:**
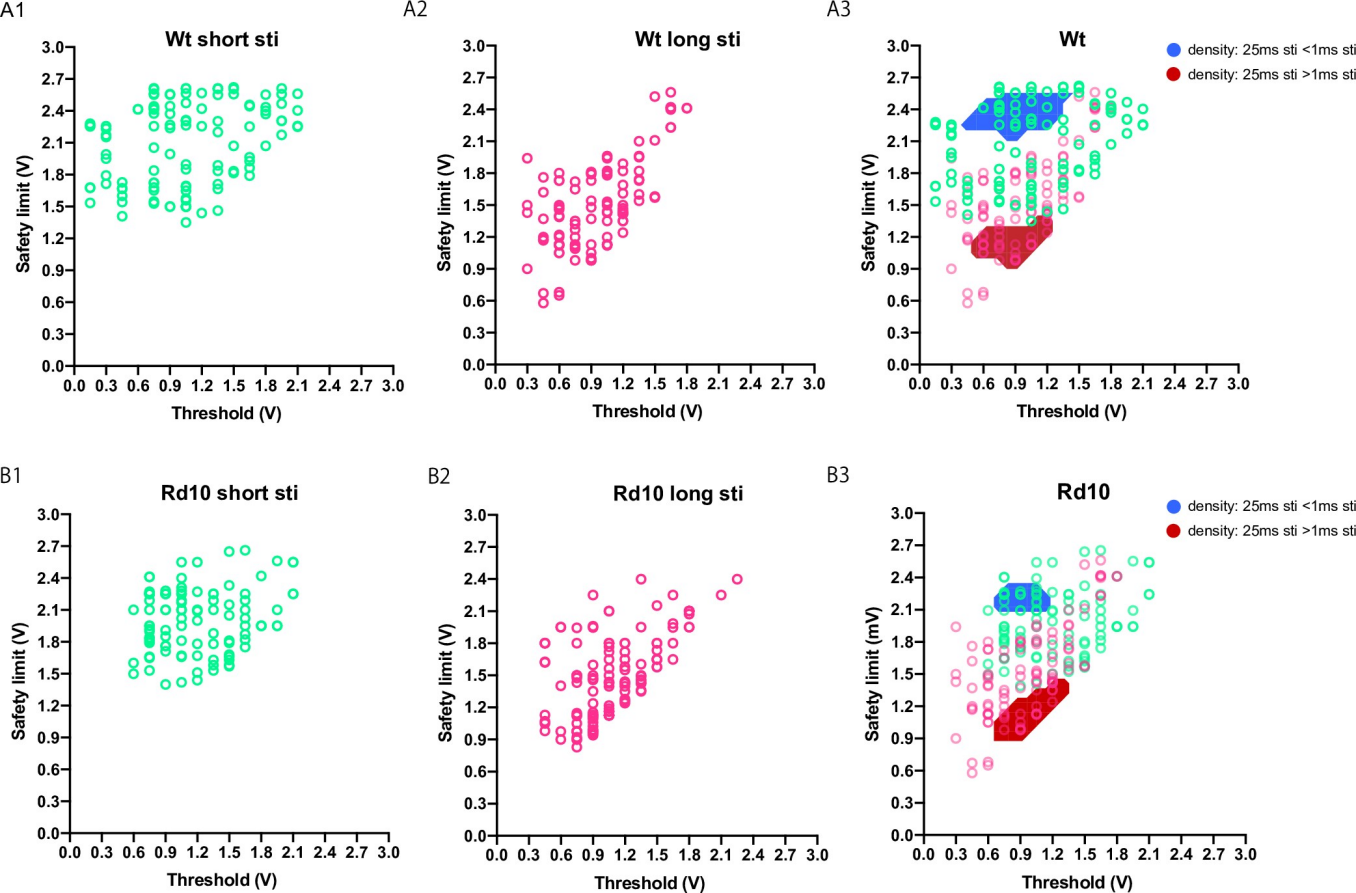
The 2-dimensional distributions of RGCs units is significantly different between the short stimulation condition and the long stimulation condition. (A1) and (A2), the scatter plot of wildtype RGC units under short pulse condition (A1) and long pulse condition (A2) with x-coordinate designating threshold and y-coordinate safety limit (see raw data in S4 Table). (A3) superposition of A1 and A2, with areas of significant density difference (p<0.05, Kernel density based local two-sample comparison test) designated by red and blue colors. (B1) and (B2), the scatter plot of rd10 RGC units under short pulse condition (B1) and long pulse condition (B2) with x-coordinate designating threshold and y-coordinate safety limit (see raw data in S4 Table). (B3) superposition of B1 and B2, with areas of significant density difference (p<0.05, Kernel density based local two-sample comparison test from the ks package in R language) designated by red and blue colors.

To reveal the relationship between the threshold and safety limit parameters under different condition, we generated a scatter plot with threshold against safety limit for each recorded RGC unit (Fig 3A1, 3A2, 3B1, and 3B2). We found that the scatter plots were significantly different between the short pulse condition and the long pulse condition (p = 3.1e-20 for the wildtype RGC units, and p = 1.1e-16 for the rd10 RGC units, Kernel density based two-sample comparison test from ks package [8] in R language [9]). The data points were significantly more concentrated along the diagonal in the long pulse condition (Fig 3A3 and 3B3, the red area, α = 0.05), but significantly more concentrated in upper left quadrant in the short pulse condition (Fig 3A3 and 3B3, the blue area, α = 0.05).

**Fig 4:**
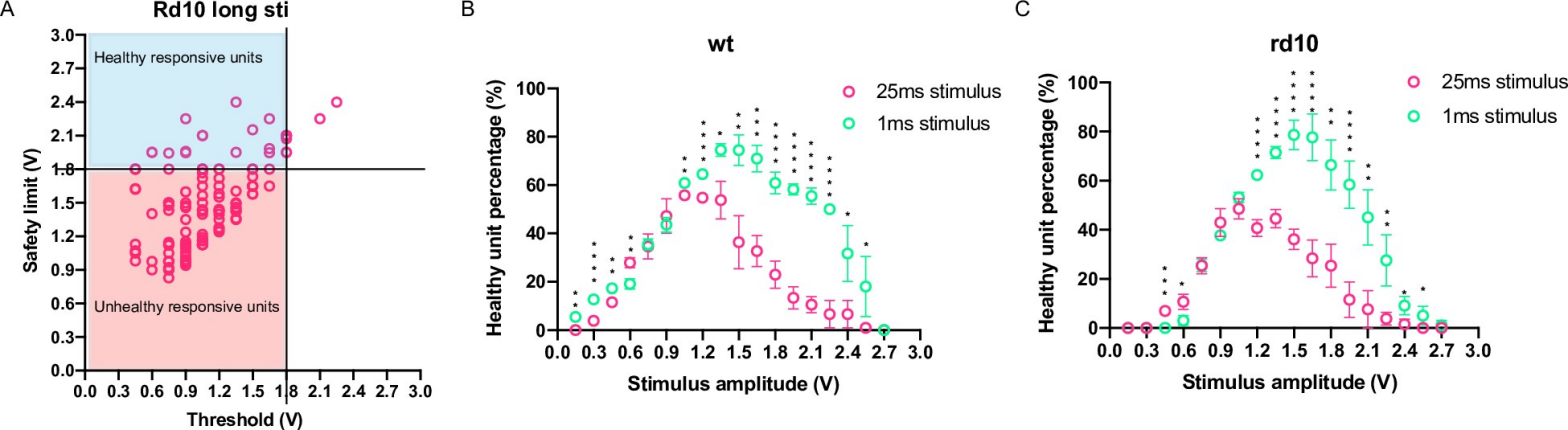
Long pulse stimulation increased RGC damaging risk for both the wildtype and rd10 RGC units using the single parameter set approach. (A) a diagram showing how the healthy responsive unit number were counted (the units in the blue area). (B) the proportion of healthy responsive unit in total wildtype RGC units against stimulation amplitude under 1ms pulse width condition and 25ms pulse width condition. (C) the proportion of healthy responsive unit in total rd10 RGC units against stimulation amplitude under 1ms pulse width condition and 25ms pulse width condition.*, p<0.05; **, p<0.0021; ***, p<0.0002; ****, p<0.0001; multiple t-test statistics were performed via false discovery rate approach, with two-stage step-up method (false discovery rate 1%, see statistic result in S5 and S6 Tables).

Using this scatter plot, we can easily count the number of units that were activated by a certain stimulus amplitude, and the number of units that stayed within safety limit. The former increased with stimulus amplitude, but the latter decreased with stimulus amplitude. To maximize the number of responsive healthy units—those with threshold parameter lower than the stimulus amplitude being tested and safety limit parameter higher than the stimulus amplitude being tested, we counted the number of responsive healthy units under different stimulus amplitude (Fig 4B and 4C). To our surprise, the long pulse stimulation preserved many less responsive healthy units than the short pulse stimulation did: for the wildtype, the maximum responsive-healthy-unit percentage that short pulse stimulation can preserve is 74±2% (4 mice), whereas the maximum responsive-healthy-unit percentage that long pulse stimulation can preserve is only 55±1% (4 mice). This phenomenon was especially conspicuous for the rd10 mouse retina: the maximum responsive-healthy-unit percentage that short pulse stimulation can preserve was 78±6% (4 mice), whereas the maximum responsive-healthy-unit percentage that long pulse stimulation can preserve was only 48±4% (5 mice). These results demonstrated that if a single parameter set (amplitude and pulse width) were optimized for all electrodes, by adopting such parameters, a significant proportion of stimulated RGC units would either be overloaded or stay dormant (24% on average for 1ms pulse condition, 49% on average for 25ms pulse condition). The expectation that every electrode would function appropriately by adopting the same optimized parameters is not practical, especially if the stimulation pulse width reached 25ms per phase, since the maximum proportion of responsive healthy RGC units under 25ms pulse condition was only ~50% for both wildtype and rd10 mice. In addition, during the stimulation ramping process, the 25ms pulse always generated more activity-decaying (unhealthy) units than the 1ms pulse at most of the stimulus amplitudes tested (S2 Fig). This was in concordance with the observation that in long pulse condition a lot more RGC units failed to recover activity one hour after the last stimulation than that in short pulse condition (65% vs 37% on average, see Table 1). These observations indicated that long pulse-width stimulus inflicted more strain on RGCs as the stimulus amplitude increased.

**Fig 5:**
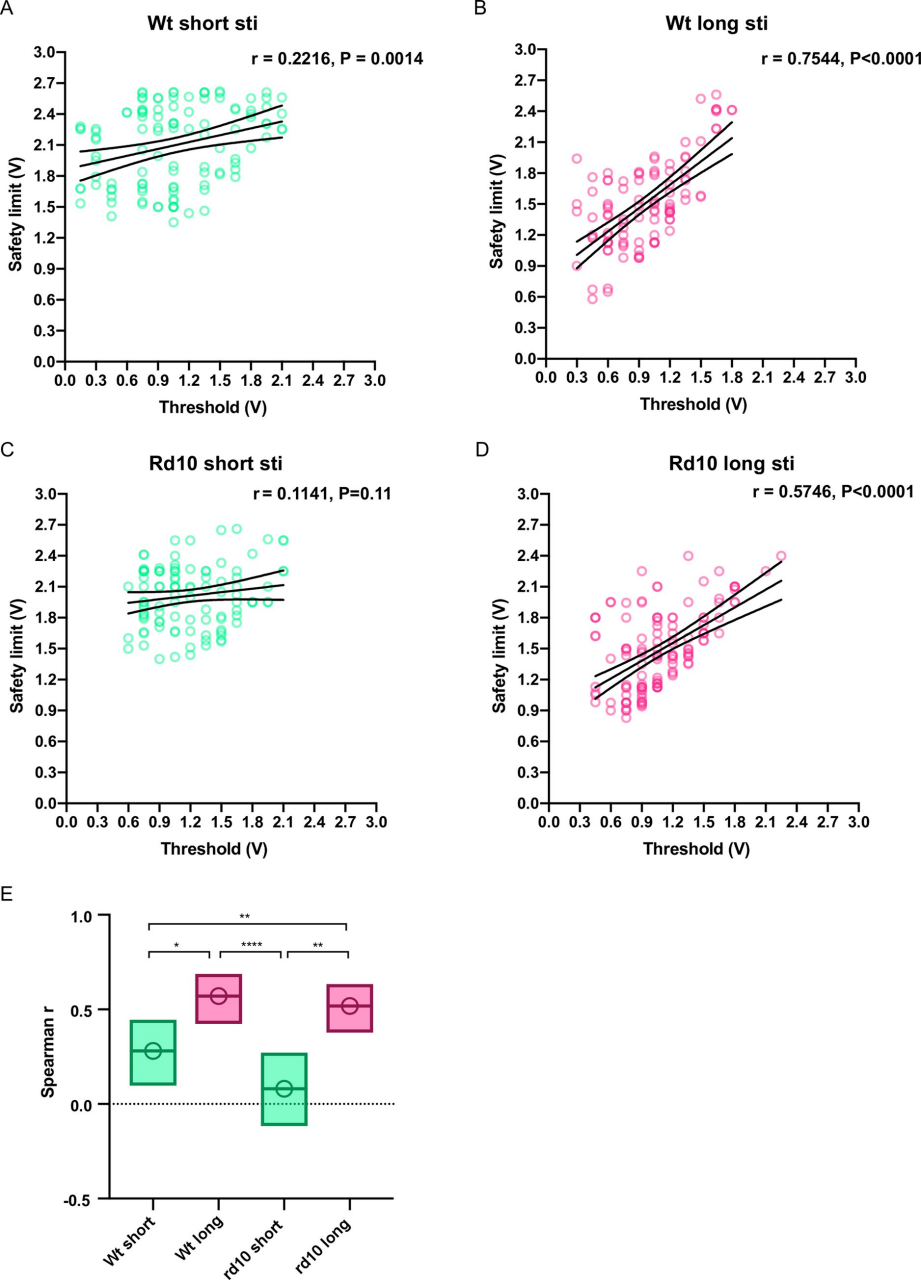
The correlation between the threshold and safety limit parameters was more apparent for the long stimulation condition. (A), (B), (C), and (D), linear regression showing the relationship between threshold and safety limit under different combinations of mouse strains and stimulation pulse-width condition. The slopes were steeper in the long pulse condition than that in the short pulse condition (see statistic result in S7–S11 Tables). (E) Spearman’s rank correlation coefficient was significantly larger in the long pulse condition than that in the short pulse condition.*, p<0.05; **, p<0.0021; ***, p<0.0002; ****, p<0.0001; statistics were performed via Fisher transformation and z-test corrected by Benjamini–Hochberg procedure (see statistic result in S12–S16 Tables).

How can we explain long pulse stimulation increased the risk of RGC damaging? From kernel density-based comparison (Fig 3A3 and 3B3), we already sensed that under long stimulation condition, the threshold and safety limit parameters were highly correlated. Linear regression of these two parameters confirmed our suspect (Fig 5A to 5D): the slopes were larger in the long pulse condition (r = 0.7544 for wildtype units, r = 0.5746 for rd10 units) than the short pulse condition (r = 0.2216 for wildtype units, r = 0.1141 for rd10 units)). The difference reached significance (p = 3.8e-8 for wildtype group, p = 5.2e-5 for rd10 group, Fisher transformation and z-test). Spearman correlation coefficient also confirmed that under long pulse condition, the threshold and safety limit parameters were significant more correlated than those under short pulse condition (Fig 5E). Also, as shown in Fig 2D, the stimulation amplitude range (the difference between safety limit and threshold) was significantly smaller under long pulse condition. All these results indicated that under long pulse condition, increase in threshold parameter only caused a corresponding (close to 1:1) increase in safety limit, which was too small to buffer the increase of stimulus amplitude; thus, while increasing stimulus amplitude can cause an increment in activated RGC number, such increment will be offset by the decrement of healthy RGC number. This would also argue that, due to stimulation pulse elongation (in our case a 25 × magnification), the stimulation intensity (pulse width × amplitude) would easily surpass the limit of RGC endurance.

In actual application of retinal prosthesis, multiple electrode will be activated simultaneously. Can multi-electrode-stimulation generate results different from that of single-electrode stimulation experiments? We did find that the cross talk between neighboring electrodes increased the electrode’s probability of activating RGCs (e.g., about 20% of the recorded RGC units can be activated by the nearby electrodes 100μm apart, see S3 Fig), so we expect multi-electrodes stimulation will lower the threshold for RGC activation. Indeed, when using a 3×3 electrodes array to stimulate RGCs, the threshold of center electrodes were indeed lowered compare to that of single-electrode stimulation (but no statistics were mentioned) [10]. In [10], the evoked activity of center electrodes also decayed as the stimulus amplitude became extreme, which was consistent with our results. To verify if multi-electrodes stimulation’s effect was different from that of single-electrode stimulation, we also used an assembly of a 3×3 electrodes array to stimulate RGCs and analyzed the signal from the central electrode (10 RGC units from 2 retinas of 2 wildtype mice for 1ms pulse condition, and 9 RGC units from 2 retinas of 2 wildtype mice for 25ms pulse condition). The stimulus pulse width was fixed at 1ms/phase and 25ms/phase, and the stimulus amplitudes were increased from 0.15V to 2.7V with a 0.15V increment step. We did find that in the 25ms pulse condition, the threshold of 3×3 electrodes stimulation was significantly lowered compare to those of single electrode stimulation (S4A Fig). In addition, we still found that long pulse condition significantly lowered the safety limit and stimulation-amplitude-range parameters compared to short pulse condition (S4B and S4C Fig), and preserved a responsive-healthy-unit proportion of only 44% at best(S4D Fig, red symbol). In comparison, the 1ms pulse width preserved a responsive-healthy-unit proportion as high as 80% (S4D Fig, green symbol). Thus, the cross-talk between electrodes did not change the results derived from single-electrode stimulation. Therefore, we conclude that if we don’t tweak each electrode’s parameter individually, i.e., we optimize a single set of parameters for all electrodes, by adopting such parameters, a significant proportion of stimulated RGCs will be inappropriately stimulated, especially under the 25ms pulse-width condition.

## Discussion

What is optimal stimulation protocol for implanted epi-retinal prosthesis? Previous studies have shown that long stimulus pulse width can improve the spatial resolution of epi-retinal prosthesis stimulation [3, 11], and it seemed essential to assimilate this discovery into stimulation parameter optimization. Nevertheless, the potential effect of elongated stimulation pulse on retinal health was not investigated. In this study, we compared the difference of stimulated RGCs’ firing rates under two pulse width conditions: a short pulse condition (1ms/phase), and a long pulse condition (25ms/phase). We observe that RGCs’ evoked response diminished during stimulation ramping, and this attenuation of firing rate were possibly irreversible and not due to refractory period, because 51% of the stimulated RGC units did not show any spontaneous spikes even after one-hour post-stimulation (Table 1). We demonstrated that under long pulse condition, the stimulation amplitude ranges RGCs can withstand is drastically compressed (Fig 2D). This suggested that when long stimulus pulse width was applied, the initial stimulus amplitude and increment of amplitude must be chosen carefully, or else the stimulation may easily surpass the limit of RGC endurance. We also demonstrated that when a single set of parameters were optimized for all electrodes, by adopting such parameters, a significant proportion of stimulated RGC units were ineffectively or inappropriately stimulated, especially in long pulse condition (about 50% in this case, Fig 4B and 4C).

Current stimulation optimization for epi-retinal prosthesis such as Argus II relies on oral report of the patients; medical staff had to optimize parameters for each microelectrode. If using long stimulation pulse (~25 ms per phase) instead of the short pulse (0.46ms) Argus II currently used, not only the initial amplitude, but also the amplitude increment, should be kept small, which meant the parameter optimization process will be more consuming, as indicated in our study. The Argus II have 60 electrodes, and it usually takes dozens of hours to finish the optimization process under default condition. It is conceivable that elongation of stimulation can extend this process to days. With the advent of high-density microelectrode array (e.g., 120 × 120) in retinal prosthesis, the task of optimizing stimulation will become extremely formidable accordingly. So, when stimulus pulse width is to be elongated, the drastic elevation of time and human resource cost must be considered.

In our study, voltage instead of current stimulation was used. This was because the minimum current amplitude step of the MEA2100 system is 1 μA; under 25 ms/phase pulse width condition, no RGC can withstand the any stimulation intensity larger than 2 μA × 25 ms in our study. It is difficult to determine the charge injected in such mode. Voltage stimulation can cause huge capacitive current at the onset of the pulse, which may provide enough charge to activate RGCs. Still, the charge holds for the remaining pulse width, and prolonged contact with such charged surface of electrodes may be detrimental to RGCs; furthermore, in the voltage stimulation mode, long stimulation pulse (at least the cathodic phase) can lock RGCs in a prolonged hyperpolarization state, which will cause excitotoxicity to RGCs. These may explain why long stimulation made RGCs vulnerable to extreme stimulus amplitudes.

How to effectively stimulate while avoid overloading RGCs in retinal prosthesis is still under heavy investigating. To optimize the parameters of electrical stimulation for epi-retinal prosthesis, an in vivo indicator for stimulated RGCs’ health state could be introduced. However, currently such indicators are barely available. Apoptosis and necroptosis markers are not desirable because they already indicate an irreversible death process, thus are not helpful to show the sub-healthy state. Small soluble fluorescent molecules can be used to reveal the damaged cells for their membrane can be ruptured by extreme electrical stimulation [12], but since the RGC somas are sheathed by Müller cells endfeet, these fluorescent molecules may not access RGC somas easily when the damage is not severe enough to dislodge the Müller cell endfeet. Optical coherence tomography has been used successfully to monitor the macro-structural change of retina after epi-retinal implant stimulation [12], and it may serve as an valuable method to monitor retinal state. Also, RGCs’ ability to fire action potential can be compromised by extreme electrical stimulation due to refractory period and calcium toxicity, so it is reasonable to closely monitor RGCs’ evoked response to increased stimulus intensity; as soon as the evoked response show sign of compromise, the stimulus intensity will be adjusted accordingly. We employed this strategy in this study. Based on this idea, future retinal prosthesis can incorporate the action-potential recording module in vivo, and display the status of stimulated RGCs by monitor their electrical activity. This can even be used to optimize the stimulation parameters for each electrode of MEA in real time in an unsupervised manner. It provides a universal indicator for stimulation efficiency and accuracy, which is independent on oral report, thus it can greatly boost preclinical research for retinal prosthesis stimulation. It may also significantly relieve the burden from medical staff and patients.

Of course, the change of evoked RGCs’ firing rate is still a preliminary indicator of RGCs’ health, but for retinal prosthesis to monitor RGCs’ health state *in vivo*, it may be one of the most convenient and realistic measures. In our study, the relation between evoked RGCs’ firing rate change and RGCs’ health was not systematically investigated, which will be addressed in our future research.

## Conclusions

Optimizing stimulation protocol is essential for clinical application of retinal prosthesis. Elongating stimulation pulse width (~25ms /phase) has been proposed as an effective method to improve spatial resolution of epi-retinal implants. However, it is unknown whether elongated stimulus pulse width will increase the risk of damaging retina. Base on a biosafety measure (RGCs’ evoked action potential number in response to electrical stimulation), we found that long pulse width significantly compressed the stimulation amplitude range RGCs can withstand, especially in the rd10 mice, which implicated that the operation window for parameter adjustment under long stimulation pulse condition was severely limited. When a single set of parameters were optimized for all electrodes, by adopting such parameters, a significant proportion of recorded RGC units were ineffectively or inappropriately stimulated, especially under the long pulse condition. We proposed that long stimulus pulse width could easily lead to reduced retinal response and thus highlighted the importance of carefully setting the stimulation amplitude in this case.

## Supporting information

S1 Fig Experiment design of the current study. (PDF)

S2 Fig The proportion of activity-decaying (unhealthy) units against stimulation amplitude in wildtype and rd10 mice under 1ms pulse condition and 25ms pulse condition. A, for wildtype RGCs. B, for rd10 RGCs. *, p<0.05; **, p<0.0021; ***, p<0.0002; ****, p<0.0001; multiple t-test statistics were performed via false discovery rate approach, with two-stage step-up method (false discovery rate 1%, see statistic result in S17 and S18 Tables).
(PDF)

S3 Fig Percentage of wildtype RGCs responding to electrical stimulation at each interelectrode distance between stimulating electrode and recording electrode with stimulus amplitude set at 1.05V, pulse width set at 25ms. (PDF)

S4 Fig A, B, and C, Threshold, safe-limit and stimulation-amplitude-range parameters of center-electrode-RGC-units from a 3×3 electrode-array when such 3×3 electrode-array was used to stimulate RGCs. D. In the 3×3 electrode-array stimulation scenario, the proportion of responsive healthy units from the center electrodes against stimulation amplitude under 1ms pulse and 25ms pulse condition. Corresponding to Figs 2B, 2C, 2D and Fig 4B. *, p<0.05; **, p<0.0021; ***, p<0.0002; ****, p<0.0001. In A, B, and C, statistics were performed via Kruskal-Wallis test and Dunn’s multiple comparison test. In D, multiple t-test statistics were performed via false discovery rate approach, with two-stage step-up method (false discovery rate 1%). See statistic result in S20, S21, S22 and S23 Tables.
(PDF)

S1 Table Kruskal-Wallis test of threshold distribution of different groups of RGC units. (CSV)

S2 Table Kruskal-Wallis test of safety limit distribution of different groups of RGC units. (CSV)

S3 Table Kruskal-Wallis test of stimulation-amplitude range distribution of different groups of RGC units. (CSV)

S4 Table The threshold and safety limit parameters of RGC units under every combination of mouse strains and stimulation pulse width. (XLSX)

S5 Table Multiple t-test for difference between wildtype responsive-healthy-RGC-unit proportions under short and long pulse conditions at different stimulation amplitude. (CSV)

S6 Table Multiple t-test for difference between rd10 responsive-healthy-RGC-unit proportions under short and long pulse conditions at different stimulation amplitude. (CSV)

S7 Table Linear regression between threshold and safety limit of RGC units in the wildtype short pulse stimulation group. (CSV)

S8 Table Linear regression between threshold and safety limit of RGC units in the wildtype long pulse stimulation group. (CSV)

S9 Table Linear regression between threshold and safety limit of RGC units in the rd10 short pulse stimulation group. (CSV)

S10 Table Linear regression between threshold and safety limit of RGC units in the rd10 long pulse stimulation group. (CSV)

S11 Table Linear regression correlation coefficient comparison. (XLSX)

S12 Table Spearman’s rank correlation coefficient between threshold and safety limit of RGC units in the wildtype short pulse stimulation group. (CSV)

S13 Table Spearman’s rank correlation coefficient between threshold and safety limit of RGC units in the wildtype long pulse stimulation group. (CSV)

S14 Table Spearman’s rank correlation coefficient between threshold and safety limit of RGC units in the rd10 short pulse stimulation group. (CSV)

S15 Table Spearman’s rank pulse correlation coefficient between threshold and safety limit of RGC units in the rd10 long stimulation group. (CSV)

S16 Table Spearman correlation coefficient comparison between different groups of RGC units. (XLSX)

S17 Table Multiple t-test for difference between wildtype unhealthy-RGC-unit proportions under short and long pulse conditions at different stimulation amplitude. (CSV)

S18 Table Multiple t-test for difference between rd10 unhealthy-RGC-unit proportions under short and long pulse conditions at different stimulation amplitude. (CSV)

S19 Table The threshold and safety limit parameters of central-electrode RGC units under every combination of mouse strains and stimulation pulse width, when a 3×3 electrode-array were used to stimulate RGCs. (XLSX)

S20 Table Kruskal-Wallis test of threshold distribution of different groups of RGC units in S19 Table. (CSV)

S21 Table Kruskal-Wallis test of safe limit distribution of different groups of RGC units in S19 Table. (CSV)

S22 Table Kruskal-Wallis test of stimulation-amplitude-range distribution of different groups of RGC units in S19 Table. (CSV)

S23 Table Multiple t-test for difference between responsive-healthy-RGC-unit proportions (at center electrode) under short and long pulse stimulation conditions at different stimulation amplitude, when a 3×3 electrode-array were used to stimulate RGCs. (CSV)
