# Supplementary material for: Optimization of stimulation parameters for epi-retinal implant based on biosafety consideration
Source: PLoS One. 2020 Jul 22;15(7):e0236176. doi: 10.1371/journal.pone.0236176 (PMC7375526; doi:10.1371/journal.pone.0236176)
Supplement: S1 Fig — (PDF) [file pone.0236176.s001.pdf]

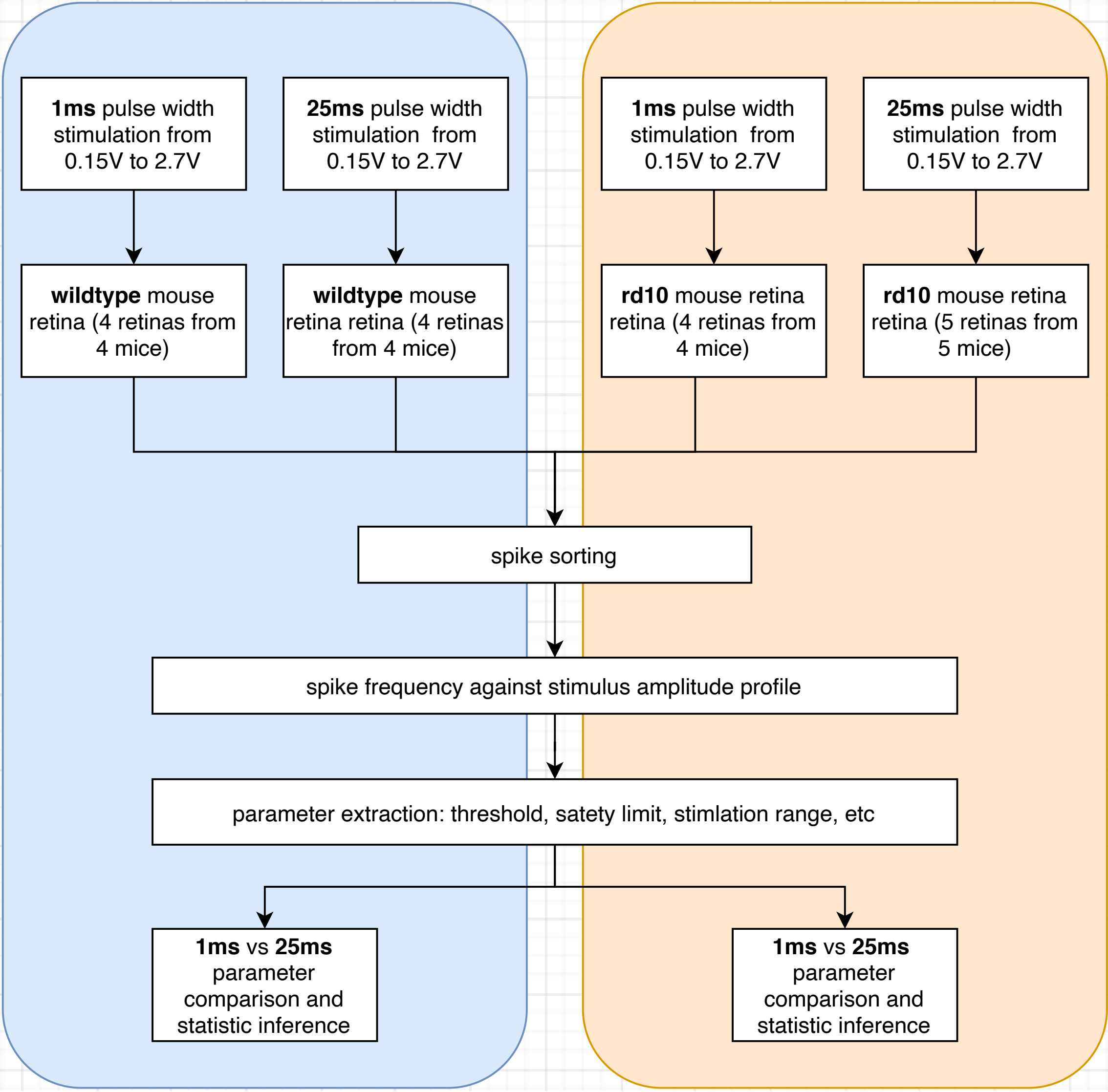
1ms pulse width stimulation from 0.15V to 2.7V
25ms pulse width stimulation from 0.15V to 2.7V
1ms pulse width stimulation from 0.15V to 2.7V
25ms pulse width stimulation from 0.15V to 2.7V
wildtype mouse retina (4 retinas from 4 mice)
wildtype mouse retina retina (4 retinas from 4 mice)
rd10 mouse retina retina (4 retinas from 4 mice)
rd10 mouse retina retina (5 retinas from 5 mice)
spike sorting
spike frequency against stimulus amplitude profile
parameter extraction: threshold, satety limit, stimlation range, etc
1ms vs 25ms parameter comparison and statistic inference
1ms vs 25ms parameter comparison and statistic inference
