# Supplementary figures and images for: Optimization of stimulation parameters for epi-retinal implant based on biosafety consideration
Source: PLoS One. 2020 Jul 22;15(7):e0236176. doi: 10.1371/journal.pone.0236176 (PMC7375526; doi:10.1371/journal.pone.0236176)

A

## wildtype unhealthy units

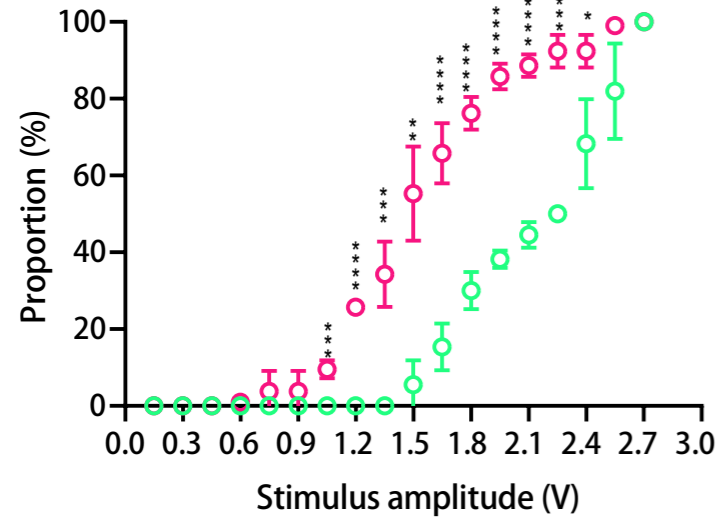

B

## rd10 unhealthy units

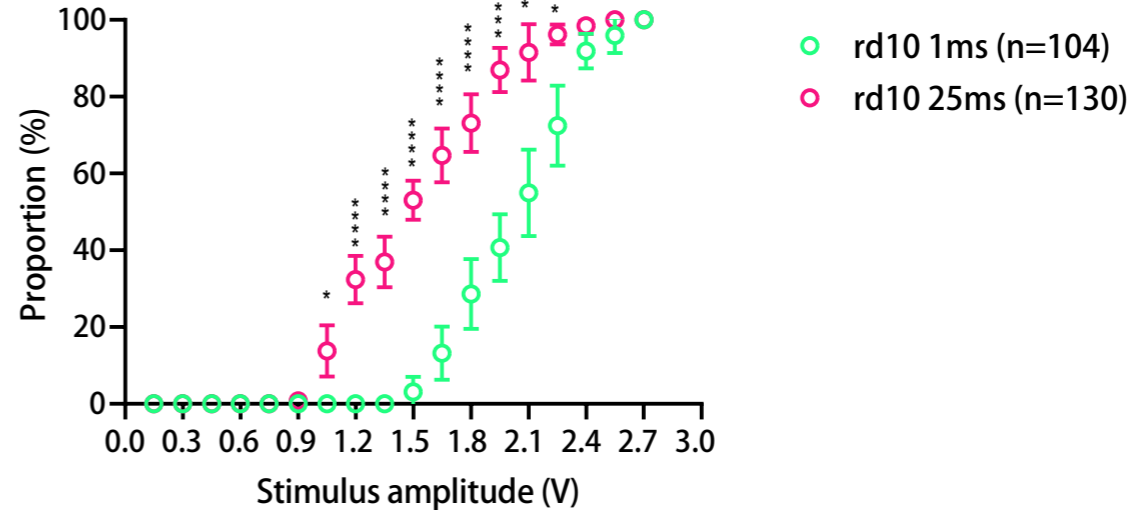

Supplement: S2 Fig — A, for wildtype RGCs. B, for rd10 RGCs. *, p<0.05; **, p<0.0021; ***, p<0.0002; ****, p<0.0001; multiple t-test statistics were performed via false discovery rate approach, with two-stage step-up method (false discovery rate 1%, see statistic result in S17 and S18 Tables). (PDF) [file pone.0236176.s002.pdf]

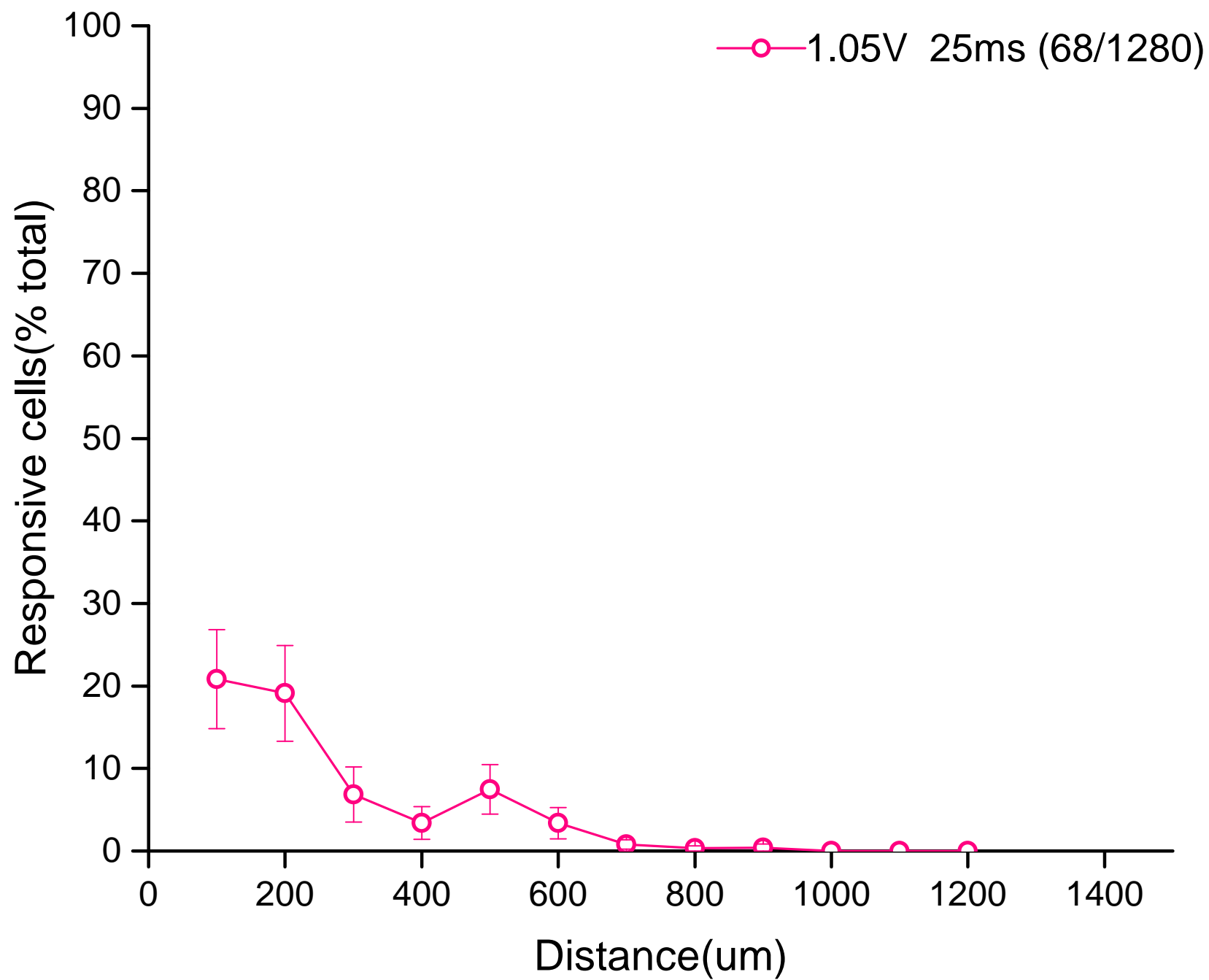

Supplement: S3 Fig — (PDF) [file pone.0236176.s003.pdf]
