# Supplementary material for: Optimization of stimulation parameters for epi-retinal implant based on biosafety consideration
Source: PLoS One. 2020 Jul 22;15(7):e0236176. doi: 10.1371/journal.pone.0236176 (PMC7375526; doi:10.1371/journal.pone.0236176)
Supplement: S4 Fig — A, B, and C, Threshold, safe-limit and stimulation-amplitude-range parameters of center-electrode-RGC-units from a 3×3 electrode-array when such 3×3 electrode-array was used to stimulate RGCs. D. In the 3×3 electrode-array stimulation scenario, the proportion of responsive healthy units from the center electrodes against stimulation amplitude under 1ms pulse and 25ms pulse condition. Corresponding to Figs 2B, 2C, 2D and Fig 4B. *, p<0.05; **, p<0.0021; ***, p<0.0002; ****, p<0.0001. In A, B, and C, statistics were performed via Kruskal-Wallis test and Dunn’s multiple comparison test. In D, multiple t-test statistics were performed via false discovery rate approach, with two-stage step-up method (false discovery rate 1%). See statistic result in S20, S21, S22 and S23 Tables. (PDF) [file pone.0236176.s004.pdf]

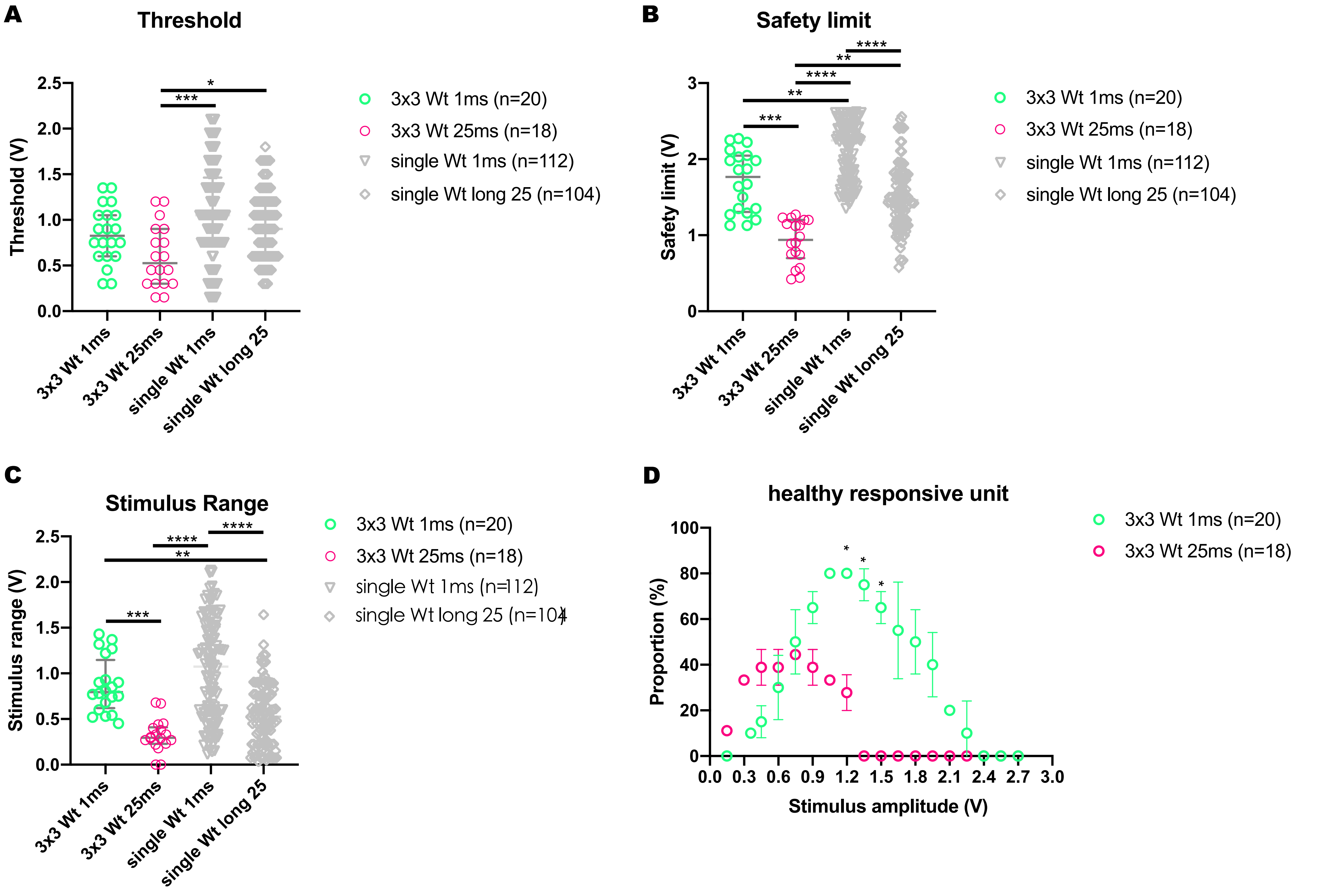
A
Threshold
Threshold (V)
3x3 Wt 1ms
3x3 Wt 25ms
single Wt 1ms
single Wt long 25
3x3 Wt 1ms (n=20)
3x3 Wt 25ms (n=18)
single Wt 1ms (n=112)
single Wt long 25 (n=104)
B
Safety limit
Safety limit (V)
C
Stimulus Range
Stimulus range (V)
D
healthy responsive unit
Proportion (%)
Stimulus amplitude (V)
